# Next-generation sequencing-based molecular diagnosis of 35 Hispanic retinitis pigmentosa probands

**DOI:** 10.1038/srep32792

**Published:** 2016-09-06

**Authors:** Qi Zhang, Mingchu Xu, Jennifer D. Verriotto, Yumei Li, Hui Wang, Lin Gan, Byron L. Lam, Rui Chen

**Affiliations:** 1 College of Life Sciences, Zhejiang University, Hangzhou, Zhejiang, China; 2 Institute of Life Sciences, Hangzhou Normal University, Hangzhou, Zhejiang, China; 3 Human Genome Sequencing Center, Baylor College of Medicine, Houston, Texas, United States; 4 Department of Molecular and Human Genetics, Baylor College of Medicine, Houston, Texas, United States; 5 Bascom Palmer Eye Institute, University of Miami Miller School of Medicine, Miami, Florida, United States; 6 Structural and Computational Biology and Molecular Biophysics Graduate Program, Baylor College of Medicine, Houston, Texas, USA; 7 The Verna and Marrs Mclean Department of Biochemistry and Molecular Biology, Baylor College of Medicine, Houston, Texas, USA; 8 Program in Developmental Biology, Baylor College of Medicine, Houston, Texas, USA

## Abstract

Retinitis pigmentosa (RP) is a heterogeneous group of inherited retinal diseases. The prevalence of RP and the mutation spectrum vary across populations. Hispanic people account for approximately 17% of the United States population, and the genetic etiologies of RP of this ethnic group still remain not well defined. Utilizing next-generation sequencing (NGS), we screened mutations in known retinal disease-causing genes in an RP cohort of 35 unrelated Hispanic probands from the Miami area. We achieved a solving rate of 66% and identified 15 novel putative pathogenic mutations, including a frequent founder mutation disrupting *PRPF31* splicing. Our data show that the mutation spectrum of Hispanic RP receives a significant impact from disease-causing alleles of Spanish origin and may also contain population-specific alleles.

Retinitis pigmentosa (RP; MIM #268000) affects 1 in 3,000 to 5,000 people worldwide. It is one of the most common forms of inherited retinal degenerations^1^. RP typically starts with night blindness during the first two decades of life and progresses gradually to tunnel vision and eventual complete blindness in some patients. These clinical manifestations result from progressive dysfunction and death of rod photoreceptors followed by cone photoreceptors throughout the retina. The clinical features of RP are highly variable and may overlap with other inherited retinal degenerations, such as cone-rod dystrophy (CRD), Leber congenital amaurosis, and Usher syndrome. In addition to the clinical heterogeneity, the genetic etiologies of RP are also complex. RP may present various modes of inheritance including autosomal dominant, autosomal recessive, X-linked and digenic in rare cases^1^. To date, mutations in at least 80 genes have been associated with RP (RetNet, https://sph.uth.edu/retnet/). Therefore, accurate molecular diagnosis of RP patients is challenging but essential for better patient management and personalized treatment.

In recent years, a number of studies have applied next-generation sequencing to understand the molecular basis of human Mendelian disorders including RP^2^,^3^,^4^. Specifically, customized target capture sequencing was used to screen mutations in known disease-causing genes with high efficiency. By this method, novel disease-causing alleles as well as genotype-phenotype correlations have been identified, leading to a substantial enhancement of our understanding of allele pathogenicity, protein function and population genetics. Hence, NGS-based molecular diagnosis has been proven as a robust approach for assessing Mendelian disease on a molecular level.

Hispanic Americans are residents of the United States (US) descending from Latin America countries or Iberian peninsula^5^. They account for 17% of US population and represent a fast-growing ethnic group^6^, highlighting a need for understanding the molecular basis of genetic disorders. However, the mutation spectrum of RP in this population has not been evaluated before except in isolated cases. We collected 35 unrelated Hispanic RP probands from the Miami area and performed next-generation sequencing-based mutation screening using a panel of 226 retinal disease genes. Our data helped to solve 23 probands and revealed potential characteristics of RP mutation spectrum in this population.

## Results

### The DNA of 35 unrelated Hispanic RP probands were collected and sequenced

**Figure 1:**
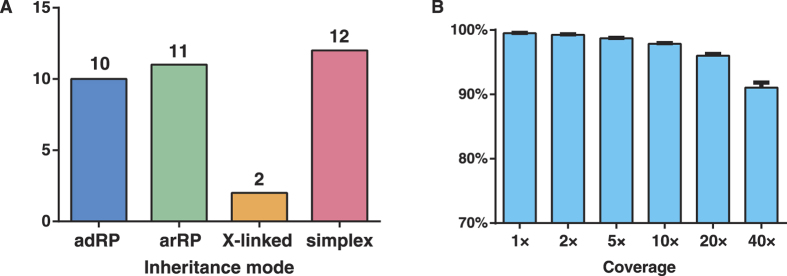
Summary of the 35 Hispanic RP probands and NGS coverage statistics. (**A**) The inheritance patterns of the probands. (**B**) The NGS coverage statistics on target regions.

A total of 35 probands with a primary diagnosis of RP were recruited in our research. Among them, there are 10 adRP cases, 11 arRP cases, 2 X-linked cases as well as 12 simplex ones (Fig. 1A). Individuals of Cuba origin account for the largest proportion (40%), followed by Colombia (14%) and Puerto Rico (14%). The probands’ DNA underwent capture enrichment and high-throughput sequencing. High quality sequencing data with an average of 91× coverage were achieved. The sequence coverage for the targeted regions is evenly distributed with evenness scores of 0.8 across all samples. Consistently, 96.0% of the targeted regions have coverage >20× and 91.1% of the targeted regions have coverage >40× (Fig. 1B). An average of 4,852 single nucleotide polymorphisms (SNPs) and 1146 small insertions and deletions (INDELs) were obtained for each sample. After filtering and annotation, an average of 16 rare variants remained.

### Putative pathogenic mutations were identified in 23 probands

To identify pathogenic variants for these probands, we applied a stepwise mutation identification strategy as previously described^3^. As shown in Table 1, putative pathogenic mutations were found in 23 probands, achieving a solving rate of 66%. In total, we identified 28 disease-causing alleles and they are listed in Table 1. Of these alleles, 13 were reported previously and 15 of them are novel. The genetic evidences of nine putative pathogenic missense variants including their frequencies and *in silico* prediction results are listed in Table 2. All putative mutations were validated by Sanger sequencing and co-segregation test was performed if DNA samples from family members were available.

### *PRPF31* is frequently mutated in the Hispanic RP cohort

**Figure 2:**
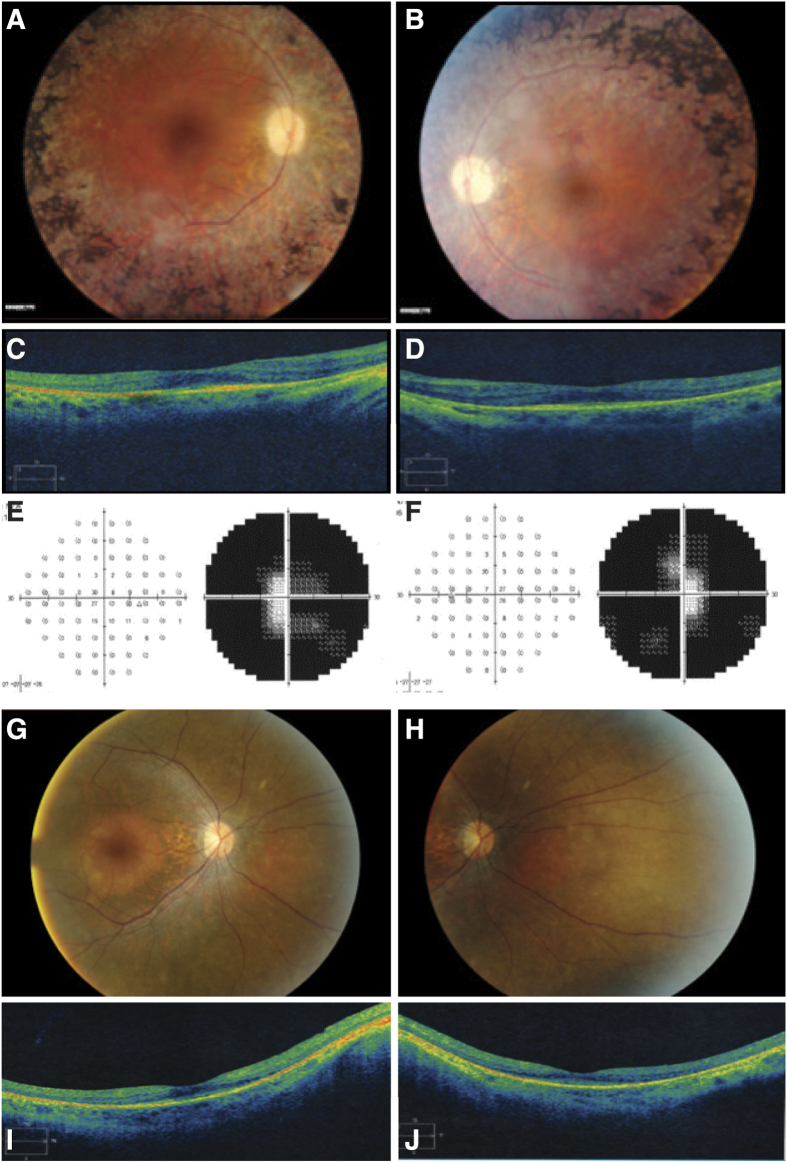
Clinical features of selected probands. Fundus images of BLM001, OD (**A**) and OS (**B**) OCT images of BLM001, OD (**C**) and OS (**D**) Visual field tests of BLM049, OD (**E**) and OS (**F**). Fundus images of BLM033, OD (**G**) and OS (**H**). OCT images of BLM033, OD (**I**) and OS (**J**).

**Figure 3:**
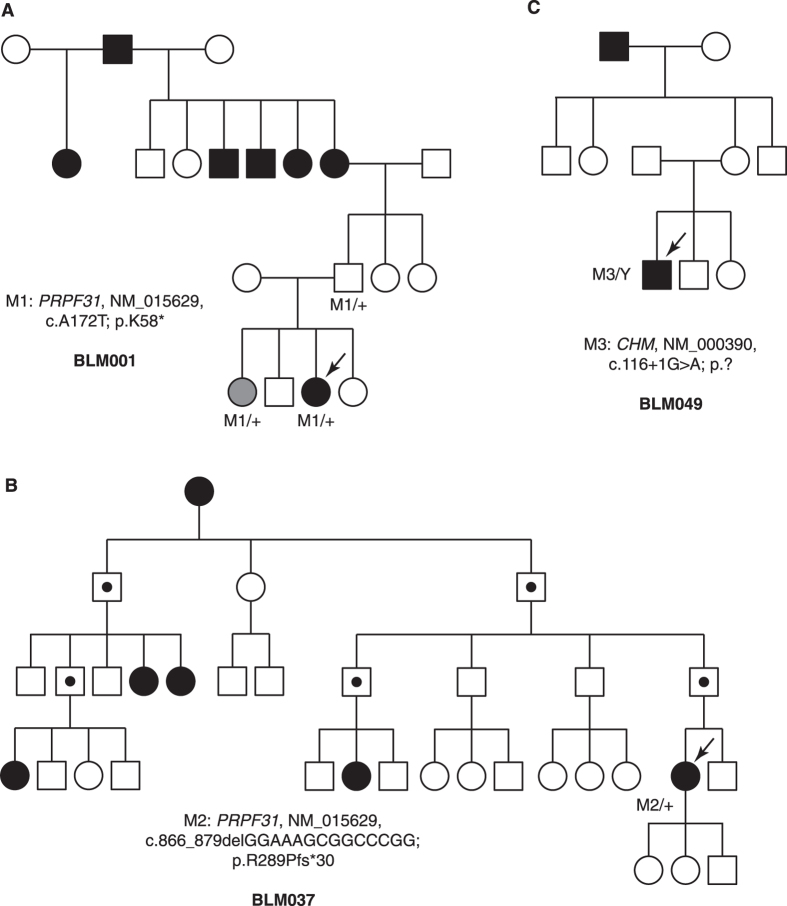
Selected family pedigrees discussed in this study. (**A**) Pedigree of adRP BLM001. The proband’s sister labeled in grey indicates mild retinal phenotype. (**B**) Pedigree of adRP BLM037. Individuals labeled with dot are obligate carriers of the *PRPF31* mutation. (**C**) Pedigree of X-linked RP BLM049 with *CHM* mutation.

For all ten adRP cases, mutations in *PRPF31*, a gene involved in pre-mRNA processing, account for five of them. The proband BLM001 (fundus and OCT images shown in Fig. 2A–D) possesses a novel *PRPF31* protein-truncating mutation (c.A172T, p.K58*). By Sanger sequencing, both the proband’s asymptomatic father and her sister with a mild retinal phenotype have this mutation (Fig. 3A), showing the incomplete penetrance and variable expressivity in this family. In proband BLM037, we identified a *PRPF31* frameshift mutation previously reported in a large Mexican family (c.866_879delGGAAAGCGGCCCGG, p.R289Pfs*30)^7^. The family in the present study is also of Mexican origin and shows a low penetrance (Fig. 3B), similar to the reported family^7^. More strikingly, as reported in the original study^7^, an apparent sex bias with females more likely being affected was observed (Fig. 3B). However, the at-risk individuals in this family are not available for detailed clinical examinations, hindering an accurate evaluation of penetrance and expressivity.

A novel splicing mutation (c.322 + 4_322 + 7delAGTG, p.?) in *PRPF31* was identified in three unrelated adRP probands (BLM067, BLM043 and BLM101). This deletion is located at the 5′ terminal of intron 5. It is predicted to disrupt the splice donor site and likely to be a loss-of-function mutation. Interestingly, all three adRP families originated from Cuba. In addition, we identified a rare variant (chr19:54632400, C > A, ExAC frequency: 9 in 25,962) 7 kb away from the *PRPF31* mutation shared by all three families, strongly suggesting that three probands share the same haplotype and this allele is likely to be a founder mutation in Cuba.

### Mutations in non-canonical RP genes were identified in four cases

In one patient BLM071, compound heterozygous missense mutations in *WDR19* were identified (c.G3533A; p.R1178Q and c.A2561C; p.K854T). The K854T mutation was novel, and the R1178Q mutation was previously reported in a patient with nephronophthisis (NPHP), polydactyly, Caroli disease and retinal dystrophy^8^. Mutations in *WDR19* are typically associated with a series of skeletal and kidney ciliopathies, while in one report, *WDR19* mutations were found in non-syndromic RP patients. We revisited this 32-year-old patient and confirmed that she has no systemic symptoms. This case further supports that *WDR19* mutations can lead to non-syndromic arRP.

RP can frequently overlap with other retinal dystrophies due to their similarities in affected tissues and disease progression. In the male proband BLM049, we identified a known hemizygous splicing mutation (c.116 + 1G > A, p.?) in *CHM*. The pedigree also appears an X-linked mode of inheritance (Fig. 3C). Mutations in *CHM* were reported to cause choroideremia, a disease that shares night blindness and progressive tunnel vision phenotype with RP. The 49-year-old proband shows severe loss of peripheral vision (Fig. 2E,F) and diffuse pigmentary retinal degeneration with macular atrophy, which is a late-stage RP phenotype that may be difficult to distinguish from late-stage choroideremia (Supplementary Figure 1). Similarly, in probands BLM033 and BLM066 (Clinical data shown in Fig. 2G–J, Supplementary Figures 2 and 3), we identified pathogenic variants in two CRD-causing genes (*C21ORF2*, *IMPG1*), and this led to clinical re-diagnosis of CRD after reassessment of the probands’ clinical phenotypes.

## Discussion

Contemporary Hispanic Americans are mainly from U.S. territorial expansion of former Spanish-speaking regions from 1819 to 1848, as well as a marked increase of immigration from Latin American countries since mid-20^th^ century^9^. According to the U.S. Census Bureau, about half of the Hispanic Americans are of European origin and over 40% are of European-African or European-Native American mixed origins^6^. Thus, the RP mutation spectrum of Hispanic Americans would in part resemble that of Europeans (particularly Spanish), as well as include certain alleles representing the distinct genetic admixture. The capture sequencing data in our study is in concordance with this hypothesis. Of all the 13 previously reported disease-causing alleles, 9 are from European studies, including 6 identified in Spanish RP families, supporting the population migration history from Spain to the Americas since the 16^th^ century. On the other hand, our data also suggest unique RP genetic etiologies in the Hispanic Americans. For example, *EYS* is a frequently mutated gene in previously reported cohorts including Spanish population^10^,^11^. However, we did not identify any case with *EYS* mutations in all 25 arRP and simplex RP cases. *PRPF31* is mutated in less than 10% of adRP cases in previous studies^12^,^13^ while in our study, *PRPF31* mutations account for 5 out of 10 adRP families. We also identified several population-specific pathogenic variants, like the *PRPF31* founder splicing mutation in Cuba-origin families and the *USH2A* variant (c.C13664T; p.P4555L) only found in Latino controls in ExAC database. These observations suggest that the genetic admixture with African and Native Americans over several centuries has resulted in an RP mutation spectrum different from that of the European/Spanish population.

One gene of interest in our study is *PRPF31*, not only because of its seemingly higher prevalence in the adRP cases, but also due to the variable penetrance and sex bias we observed. Given *PRPF31* disease-causing alleles are enriched with loss-of-function variants, we can infer that *PRPF31* mutations lead to adRP through a haploinsufficiency mechanism. Studies have shown that differential PRPF31 expression level and some genetic modifiers contribute to the incomplete penetrance or variable expressivity of *PRPF31*-associated adRP^14^,^15^,^16^. The sex bias observed in both Mexican families in our study and the previous report^7^, is likely to be specifically associated with this sub-population, or even this allele *per se*, since no other *PRPF31*-associated adRP has been found with similar phenomenon. The low penetrance and sex bias also features the particular need of genetic testing and subsequent counseling for asymptomatic disease-causing allele carriers.

Our data confirmed the contributions of mutations in non-canonical RP genes in non-syndromic RP cases. On the molecular level, RP can frequently overlap with other retinal degenerations such as CRD and Stargart disease^17^, which is reflected by mutations in *CHM*, *C21ORF2* and *IMPG1* in our study. This phenomenon has increasingly led us to rethink how the molecular diagnosis aid the clinical diagnosis in the precision medicine era. In addition, hypomorphic mutations in a dozen of syndrome-causing genes can lead to milder phenotypes (non-syndromic RP), which is shown by a list of recent WES studies^18^,^19^,^20^,^21^,^22^,^23^, and in our study, by the *WDR19* case. These studies often significantly expanded the phenotype spectrum associated with syndrome-causing genes, sometimes unexpectedly, highlighting the complicated molecular etiology underlying human retinal degenerations. Hence, we suggest that additional genes, particular those genes associated with retinal phenotypes in Mendelian disorders but with an underappreciated retinal function, need to be included in the target capture sequencing panel for revealing further genetic complexity in non-syndromic RP.

In 12 probands, we did not identify putative disease-causing variants. Several approaches may contribute to reveal the missing heritability: first, identifications of mutations in a novel disease-causing gene require whole-exome sequencing studies; second, copy number variation in known RP genes may need to be solved by higher coverage target capture sequencing or array comparative genomic hybridization (aCGH); third, the unidentified mutations in known RP genes may reside in deep intronic regions that affect pre-mRNA splicing or regulatory sequences. Future studies focusing on non-coding regions with more available whole genome sequencing control data and improved ability of variant annotation would help to unravel pathogenic mutations of this category.

In summary, to our knowledge, this study represents the first molecular diagnosis of a sizeable Hispanic RP patient cohort in the U.S. Our targeted NGS-based diagnostic approach successfully solved 23 out of 35 RP cases with the identification of 17 novel disease-causing alleles, thus improving our knowledge of RP etiology in this population. It should be noted that the genetic components of Hispanic Americans are not homogeneous and vary from differences in geographic regions and countries of ancestral origin, etc. Hence, additional molecular diagnostic studies with detailed ethnic classification would allow more accurate characterization of RP spectrum and provide better patient care in this fast-growing population in the U.S.

## Methods

### Clinical diagnosis of RP patients and sample collection

The studied cohort consisted of 35 probands and their family members recruited in the Miami area. A detailed clinical history and complete ophthalmic examination including best-corrected Snellen visual acuity, visual fields testing, slit-lamp biomicroscopy, fundoscopy and full-field electroretinogram were performed on each patient. The research was accomplished in accordance with the tenets of the declaration of Helsinki. Written informed consents were obtained from each participant or legal guardians. Peripheral blood was collected in EDTA tubes for DNA extraction. All experimental methods were approved by the Institutional Review Boards of Baylor College of Medicine and University of Miami Miller School of Medicine, and they were performed in accordance with relative guidelines and regulations.

### Library preparation and capture sequencing

Pre-capture Illumina libraries were generated as described in previous literature^24^,^25^,^26^. Briefly, 1 μg of genomic DNA was sheared into 300–500 bp fragments. The 5′ ends of the DNA fragments were phosphorylated by polynucleotide kinase and a single adenine base was added to the 3′ ends using Klenow exo-nuclease. Y-shape index adapters were ligated to the DNA fragments, and then 10 cycles of PCR amplification were applied to each sample. Finally, 300–500 bp fragments were isolated by bead purification. The pre-capture libraries were quantified by the PicoGreen fluorescence assay kit (Invitrogen, Carlsbad, CA, USA). For each capture reaction, 25–50 samples were pooled together. The targeted DNA was captured, washed and recovered using Agilent Hybridization and Wash Kits. (Agilent Technologies, Santa Clara, CA, USA). Captured DNA libraries were sequenced on Illumina HiSeq 2000 (Illumina, Inc., San Diego, CA) as 100 bp paired-end reads following the manufacturer’s protocols. The genes included in target capture panel^27^ were listed in the Supplementary Information (Supplementary Table 1).

### Bioinformatics analysis

Paired-end sequencing reads were obtained and aligned to human hg19 genome using BWA version 0.6.1. Base quality recalibration and local realignment was done by the Genome Analysis Tool Kit version 1.05974. Atlas-SNP2 and Atlas-Indel2 were used for calling SNPs and Indels. Variant frequency data were obtained from a set of public and internal control databases including Exome Aggregation Consortium (ExAC) database, CHARGE consortium^28^, ESP-6500^29^ and 1000 Genome Project^30^. Since RP is rare Mendelian disorder, variants with a frequency higher than 1/200 (for a recessive model) or 1/10,000 (for a dominant model) were filtered out. Then synonymous and deep intronic (distance >10 bp from exon-intron junctions) variants were excluded from following analysis. ANNOVAR (version 11/12/2014) and dbNSFP suite (version 2.9, contains SIFT, PolyPhen-2, LRT, MutationTaster, MutationAssessor, etc.) were used to annotate protein-altering changes. Known retinal disease-causing alleles were detected based on the HGMD professional database (version 11/15/2014).

### Sanger validation and co-segregation test

Each putative disease-causing mutation was validated by Sanger sequencing. Primer3 was used to design a pair of primers to generate amplicons that cover 500 bp region around the mutation site. The PCR amplicons were Sanger sequenced on an ABI 3730XL Genetic Analyzer. The results were analyzed by Sequencher 5.0.

## Additional Information

**How to cite this article**: Zhang, Q. *et al.* Next-generation sequencing-based molecular diagnosis of 35 Hispanic retinitis pigmentosa probands. *Sci. Rep.* **6**, 32792; doi: 10.1038/srep32792 (2016).

## Supplementary Material

Supplementary Information

## Figures and Tables

**Table 1:** Putative pathogenic variants identified in 23 tentatively solved RP probands.

Patients ID	Sex	Age	Gene	Accession ID	Genotype	Variants	Inheritance	ACMG	Previously reported?
adRP cases									
BLM091	M	38	*IMPDH1*	NM_001142575	Het	c.612_614delTTC (p.K206del)	U	LP	No
BLM067	M	27	*PRPF31*	NM_015629	Het	c.322 + 4_322 + 7delAGTG (p.?)	U	P	No
BLM043	F	19	*PRPF31*	NM_015629	Het	c.322 + 4_322 + 7delAGTG (p.?)	Mat.	P	No
BLM101	M	57	*PRPF31*	NM_015629	Het	c.322 + 4_322 + 7delAGTG (p.?)	U	P	No
BLM001	F	13	*PRPF31*	NM_015629	Het	c.A172T (p.K58*)	Pat.	P	No
BLM037	F	32	*PRPF31*	NM_015629	Het	c.866_879delGGAAAGCGGCCCGG (p.R289Pfs*30)	Pat.	P	Yes^7^
BLM066	M	47	*IMPG1*	NM_001563	Het	c.T1823C (p.L608P)	U	US	No
arRP cases									
BLM029	M	49	*ABCA4*	NM_000350	Het	c.T6179G (p.L2060R)	U	P	Yes^31^
			*ABCA4*	NM_000350	Het	c.G6089A (p.R2030Q)	U	P	Yes^32^
BLM026	M	35	*USH2A*	NM_206933	Het	c.G12575A (p.R4192H)	U	P	Yes^33^
			*USH2A*	NM_206933	Het	c.C13664T (p.P4555L)	U	US	No
BLM074	M	73	*RP1*	NM_006269	Het	c.C1625G (p.S542*)	U	P	Yes^34^
			*RP1*	NM_006269	Het	c.C4105T (p.Q1369*)	U	P	No
BLM022	F	15	*PDE6B*	NM_001145292	Homo	c.703delC (p.L235Wfs*33)	Both	P	No
BLM071	F	24	*WDR19*	NM_025132	Het	c.G3533A (p.R1178Q)	U	P	Yes^8^
			*WDR19*	NM_025132	Het	c.A2561C (p.K854T)	U	LP	No
BLM012	F	21	*RDH12*	NM_152443	Het	c.C146A (p.T49K)	Mat.	P	Yes^35^
			*RDH12*	NM_152443	Het	c.C295A (p.L99I)	Pat.	P	Yes^36^
X-linked RP cases									
BLM008	M	16	*RPGR*	NM_001034853	Hemi	c.2333delA (p.E778Pfs*83)	Mat.	P	No
BLM049	M	48	*CHM*	NM_000390	Hemi	c.116 + 1G > A (p.?)	Mat.	P	Yes^37^
simplex RP cases									
BLM088	M	58	*RHO*	NM_000539	Homo	c.C408A (p.Y136*)	U	P	Yes^38^
BLM086	M	14	*RPGR*	NM_001034853	Hemi	c.G494A (p.G165D)	U	LP	No
BLM045	F	20	*PRPF8*	NM_006445	Het	c.C5041T (p.R1681W)	U	LP	No
BLM081	M	46	*USH2A*	NM_206933	Het	c.G12575A (p.R4192H)	U	P	Yes^33^
			*USH2A*	NM_206933	Het	c.T9799C (p.C3267R)	U	P	Yes^39^
BLM053	M	32	*USH2A*	NM_206933	Het	c.1841-2A > G (p.?)	U	P	Yes^40^
			*USH2A*	NM_206933	Het	c.G8254A (p.G2752R)	U	P	Yes^41^
BLM057	F	52	*USH2A*	NM_206933	Homo	c.T12443C (p.L4148P)	U	US	No
BLM097	F	42	*PDE6B*	NM_000283	Homo	c.G704C (p.R235P)	U	LP	No
BLM033	F	36	*C21ORF2*	NM_001271440	Het	c.G218C (p.R73P)	U	US	No
			*C21ORF2*	NM_001271440	Het	c.G364C (p.D122H)	U	LP	No

Age, the age at clinical examinations; Inheritance, the paternal or maternal origin of the putative variant; ACMG, variant classification based on ACMG guidelines^42^. Het, heterozygous; Homo, homozygous; Hemi, hemizygous; Mat., maternal; Pat., paternal; U, unknown due to lack of parental DNA samples; P, pathogenic; LP, likely pathogenic; US, uncertain significance.

**Table 2:** Population frequencies and *in silico* predictions of novel missense putative pathogenic variants.

Gene	Accession ID	Variants	ExAC Frequency	SIFT	Polyphen2	LRT	MT	MA
*IMPG1*	NM_001563	c.T1823C (p.L608P)	Absent	D	PD	De	Di	M
*USH2A*	NM_206933	c.C13664T (p.P4555L)	4 in 121,378	D	PD	U	Di	M
*USH2A*	NM_206933	c.T12443C (p.L4148P)	Absent	T	PS	N	P	M
*WDR19*	NM_025132	c.A2561C (p.K854T)	Absent	T	PD	De	Di	M
*RPGR*	NM_001034853	c.G494A (p.G165D)	Absent	D	PD	De	Di	M
*PRPF8*	NM_006445	c.C5041T (p.R1681W)	Absent	D	PD	De	Di	H
*PDE6B*	NM_000283	c.G704C (p.R235P)	5 in 121,200	D	PD	De	Di	M
*C21ORF2*	NM_001271440	c.G218C (p.R73P)	37 in 110,678	T	PD	N	P	M
*C21ORF2*	NM_001271440	c.G364C (p.D122H)	Absent	D	PD	De	Di	H

SIFT, Scale-Invariant Feature Transform; Polyphen2, Polymorphism Phenotyping v2; LRT, likelihood ratio test; MT, Mutation Taster; MA, Mutation Assessor; D, damaging; T, tolerant; PD, probably damaging; PS, possibly damaging; De, deleterious; U, unknown; N, neutral; Di, disease-causing; P, polymorphism; M, medium damaging; H, high damaging.
