# Supplementary material for: Next-generation sequencing-based molecular diagnosis of 35 Hispanic retinitis pigmentosa probands
Source: Sci Rep. 2016 Sep 6;6:32792. doi: 10.1038/srep32792 (PMC5011706; doi:10.1038/srep32792)
Supplement: Supplementary Information [file srep32792-s1.pdf]

SUPPLEMENTARY INFORMATION

Next-generation sequencing-based molecular diagnosis of 35 Hispanic retinitis pigmentosa probands

Qi Zhang, Mingchu Xu, Jennifer D. Verriotto, Yumei Li, Hui Wang, Lin Gan, Byron L. Lam, Rui Chen

Supplementary Table 1. List of known retinal disease genes screened in this study.

ABCA4	*CDH3*	*GNAT2*	*MKKS*	*PLA2G5*	*SAG*
ABCC6	*CDHR1*	*GNPTG*	*MKS1*	*PRCD*	*SDCCAG8*
ABHD12	*CEP164*	*GPR179*	*MPV17*	*PRD*	*SEMA4A*
ACBD5	*CEP290*	*GRK1*	*MTTP*	*PROM1*	*SLC24A1*
ADAM9	*CERKL*	*GRM6*	*MYO7A*	*PRPF3*	*SLC6A5*
ADAMTS18	*CFB*	*GUCA1A*	*NDP*	*PRPF31*	*SNRNP200*
ADGRA3	*CFH*	*GUCA1B*	*NEK2*	*PRPF6*	*SPATA7*
ADGRV1	*CHM*	*GUCY2D*	*NMNAT1*	*PRPF8*	*TEAD1*
AHI1	*CIB2*	*HARS*	*NPHP1*	*PRPH2*	*TIMM8A*
AIPL1	*CISD2*	*HMCN1*	*NPHP3*	*PVRL1*	*TIMP3*
ALMS1	*CLN3*	*HTRA1*	*NPHP4*	*RAB28*	*TK2*
ARL2BP	*CLRN1*	*IDH3B*	*NR2E3*	*RAX2*	*TLR3*
ARL6	*CNGA1*	*IFT140*	*NRL*	*RB1*	*TLR4*
ARMS2	*CNGA3*	*IFT172*	*NYX*	*RBP3*	*TLR6*
ATXN7	*CNGB1*	*IFT27*	*OAT*	*RBP4*	*TMEM126A*
BBIP1	*CNGB3*	*IMPDH1*	*OFD1*	*RCD1*	*TMEM216*
BBS1	*CNNM4*	*IMPG1*	*OPA1*	*RD3*	*TMEM237*
BBS10	*COL11A1*	*IMPG2*	*OPA3*	*RDH12*	*TOPORS*
BBS12	*COL2A1*	*INPP5E*	*OPN1LW*	*RDH5*	*TREX1*
BBS2	*COL9A1*	*INVS*	*OPN1MW*	*RGR*	*TRIM32*
BBS4	*CRB1*	*IQCB1*	*OPN1MW2*	*RGS9*	*TRPM1*
BBS5	*CRX*	*ITM2B*	*OPN1SW*	*RGS9BP*	*TSPAN12*
BBS7	*CYP4V2*	*JAG1*	*OTX2*	*RHO*	*TTC8*
BBS9	*DFNB31*	*KCNJ13*	*PANK2*	*RIMS1*	*TTPA*
BEST1	*DHDDS*	*KCNV2*	*PAX2*	*RLBP1*	*TULP1*
C1QTNF5	*DMD*	*KIAA1549*	*PCDH15*	*ROM1*	*UNC119*
C2	*DTHD1*	*KIF11*	*PDE6A*	*RP1*	*USH1C*
C21ORF2	*EFEMP1*	*KLHL7*	*PDE6B*	*RP1L1*	*USH1G*
C2ORF71	*ELOVL4*	*LCA5*	*PDE6C*	*RP2*	*USH2A*
C3	*EMC1*	*LRAT*	*PDE6G*	*RP8*	*VCAN*
C8ORF37	*ERCC6*	*LRIT3*	*PDE6H*	*RP9*	*VPS13B*
CA4	*EYS*	*LRP1B*	*PDZD7*	*RPE65*	*WDPCP*
CABP4	*FAM161A*	*LRP5*	*PEX1*	*RPGR*	*WDR19*
CACNA1F	*FBLN5*	*LZTFL1*	*PEX2*	*RPGRIP1*	*WFS1*
CACNA2D4	*FLVCR1*	*MAK*	*PEX7*	*RPGRIP1L*	*ZNF408*
CAPN5	*FSCN2*	*MERTK*	*PGK1*	*RRM2B*	*ZNF423*
CC2D2A	*FZD4*	*MFN2*	*PHYH*	*RS1*	*ZNF513*
CDH23	*GNAT1*	*MFRP*	*PITPNM3*		

Supplementary Figure 1. The fundus photo of the *CHM* case (BLM049, OD).

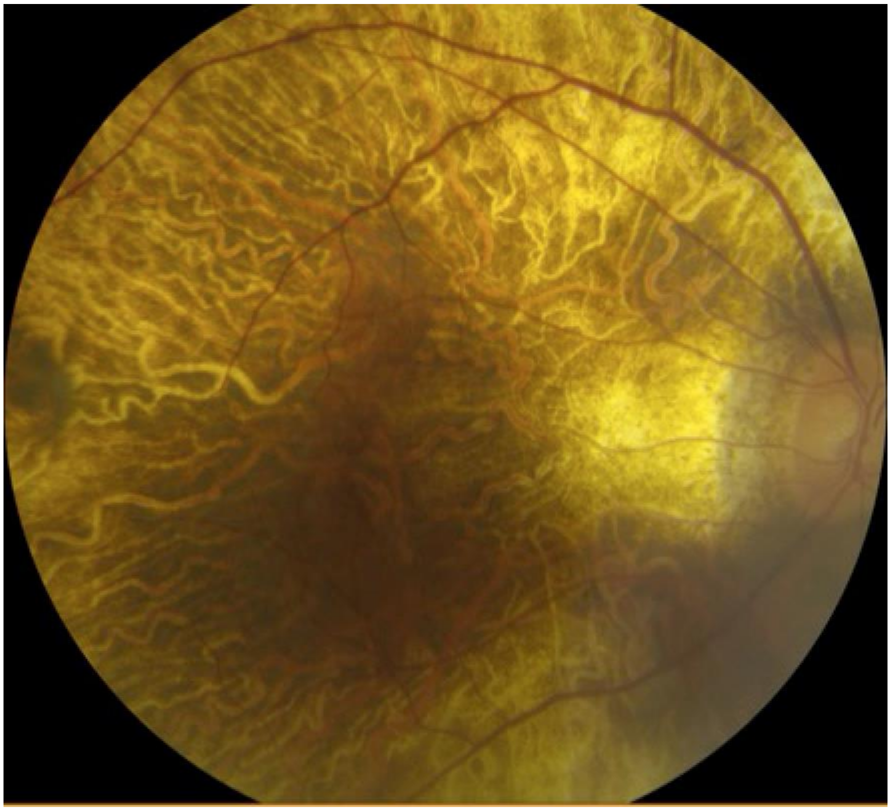

Supplementary Figure 2. The ERG data of CRD case BLM066.

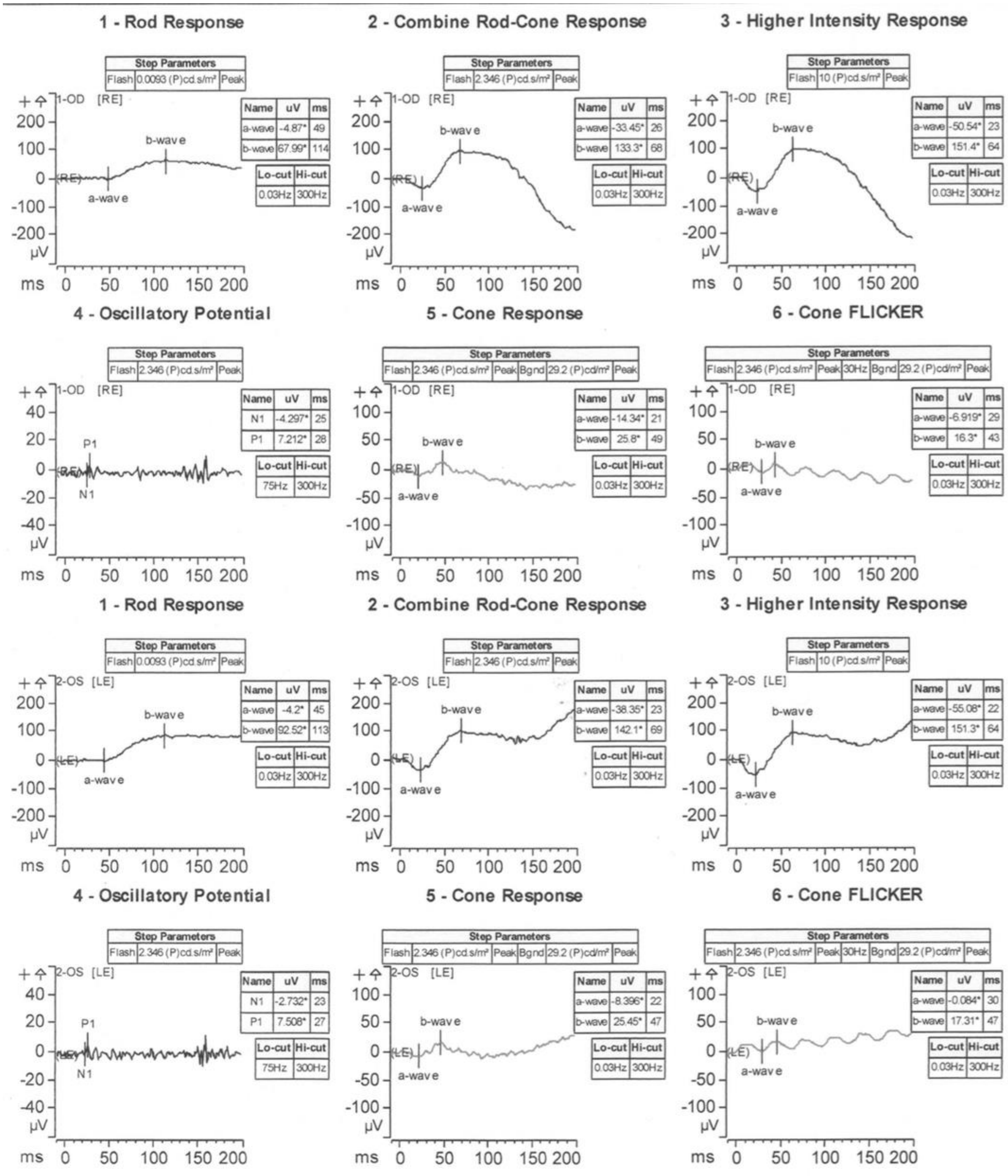

Supplementary Figure 3. The ERG data of CRD case BLM033.

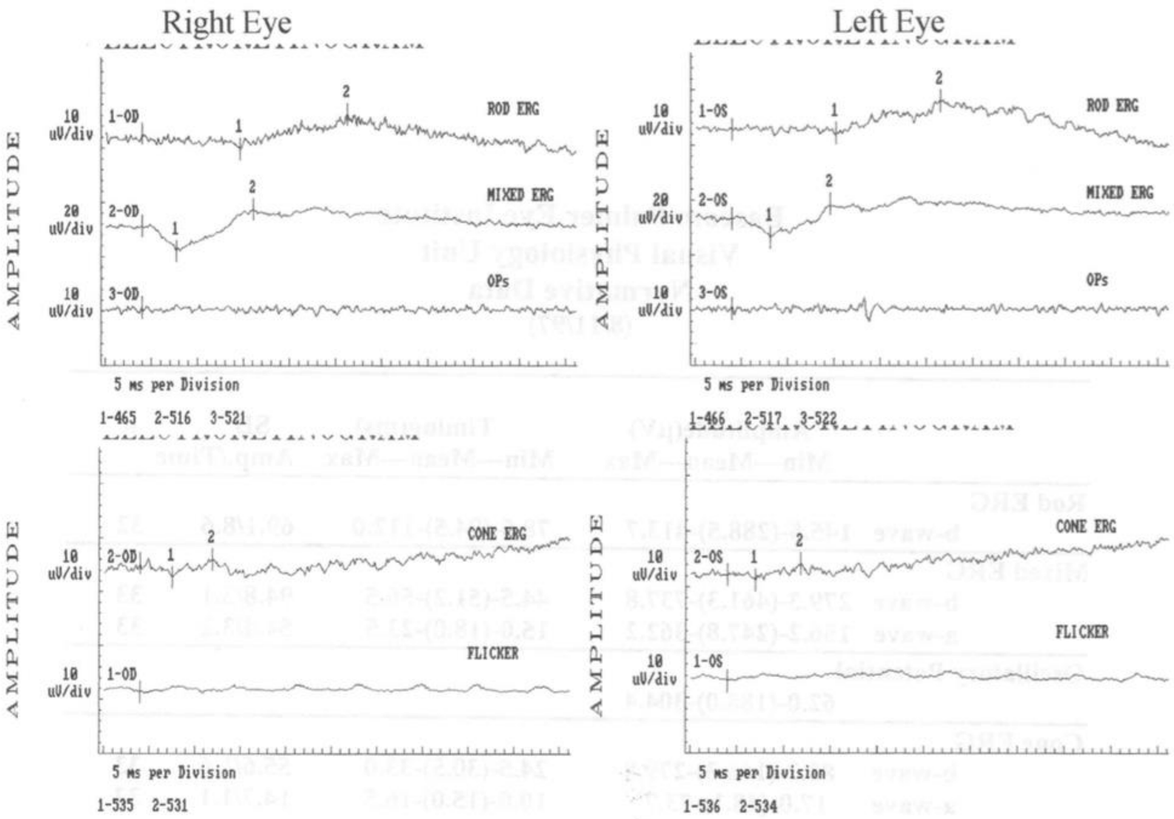

		Amplitude (uV)		Timing (ms)	
		right eye	left eye	right eye	left eye
**ROD ERG:**	**b-wave**	**32.5**	**29.9**	**111.5**	**112.5**
**MIXED ERG:**	**b-wave**	**77.9**	**62.7**	**60.5**	**53.0**
	**a-wave**	**45.2**	**39.1**	**18.5**	**20.5**
**OSCILLARY POTENTIALS:**					
**CONE ERG:**	**b-wave**	**17.6**	**15.3**	**39.5**	**39.0**
	**a-wave**	**7.9**	**5.0**	**17.5**	**15.0**
	**30 Hz flicker**	**5.3**	**4.9**	**41.1**	**40.7**
